# Carbohydrate mouth rinse failed to reduce central fatigue, lower perceived exertion, and improve performance during incremental exercise

**DOI:** 10.3389/fnut.2024.1329074

**Published:** 2024-02-20

**Authors:** Flávio O. Pires, Fabiano A. Pinheiro, Cayque Brietzke, Paulo Estevão Franco-Alvarenga, Katherine Veras, Eugênia C. T. de Matos, André L. F. Rodacki, Carlos Ugrinowitsch

**Affiliations:** ^1^Exercise Psychophysiology Research Group, School of Arts, Science and Humanities, University of São Paulo, São Paulo, Brazil; ^2^School of Physical Education and Sports, University of São Paulo, São Paulo, Brazil; ^3^Department of Nutrition, University of Mogi das Cruzes, São Paulo, Brazil; ^4^Department of Physical Education, Federal University of Paraná, Paraná, Brazil; ^5^Department of Health Sciences and Human Performance, The University of Tampa, Tampa, FL, United States

**Keywords:** cycling, endurance performance, supplementation, brain, V̇O_2MAX_

## Abstract

We examined if carbohydrate (CHO) mouth rinse may reduce central fatigue and perceived exertion, thus improving maximal incremental test (MIT) performance. Nine recreational cyclists warmed up for 6 min before rinsing a carbohydrate (CHO) or placebo (PLA) solution in their mouth for 10 s in a double-blind, counterbalanced manner. Thereafter, they performed the MIT (25 W·min^−1^ increases until exhaustion) while cardiopulmonary and ratings of perceived exertion (RPE) responses were obtained. Pre- to post-MIT alterations in voluntary activation (VA) and peak twitch torque (Tw) were determined. Time-to-exhaustion (*p* = 0.24), peak power output (PPO; *p* = 0.45), and V̇O_2MAX_ (*p* = 0.60) were comparable between conditions. Neither treatment main effect nor time–treatment interaction effect were observed in the first and second ventilatory threshold when expressed as absolute or relative V̇O_2_ (*p* = 0.78 and *p* = 0.96, respectively) and power output (*p* = 0.28 and *p* = 0.45, respectively) values, although with moderate-to-large effect sizes. RPE increased similarly throughout the tests and was comparable at the ventilatory thresholds (*p* = 0.56). Despite the time main effect revealing an MIT-induced central and peripheral fatigue as indicated by the reduced VA and Tw, CHO mouth rinse was ineffective in attenuating both fatigues. Hence, rinsing the mouth with CHO was ineffective in reducing central fatigue, lowering RPE, and improving MIT performance expressed as PPO and time-to-exhaustion. However, moderate-to-large effect sizes in power output values at VT_1_ and VT_2_ may suggest some beneficial CHO mouth rinse effects on these MIT outcomes.

## 1 Introduction

Carbohydrate (CHO) mouth rinse has been suggested as a potential aid to improve endurance performance in exercises with different durations (1, 2). The suggested mechanism underpinning the CHO mouth rinse effects on exercise performance is the likely enhanced neuromuscular activation and reduced rating of perceived exertion (RPE) (3, 4) when the CHO is sensed by oral cavity sensors (5, 6). For example, Gant et al. (6) found an increased force production in participants rinsing their mouths with CHO due to a facilitated motor output as a result of the enhanced corticospinal excitability. Others have observed that the improved cycling performance with CHO mouth rinse was associated with a reduced RPE during exercise (3). Thus, the overall suggestion is that the improved endurance motor output with CHO mouth rinse is due to an ameliorated neuromuscular activation and attenuated RPE (6).

Most studies reporting an improved endurance cycling performance with CHO mouth rinse have used trials lasting more than 30–60 min (2). These longer-lasting trials are characterized by submaximal power output values, that is, values usually ≤80% of the peak power output (PPO) attained in maximal incremental test (MIT), therefore eliciting central rather than peripheral fatigue (7). However, a previous study (8) observed that CHO mouth rinse failed to improve performance in a shorter-lasting cycling time trial (4 km distance), eliciting mean power output values closer to PPO values (i.e., ≥80% PPO) as peripheral rather than central fatigue would limit this type of effort (7, 9). Together, these results may suggest that CHO mouth rinse has the potential to improve endurance cycling performance during exercises requiring power output values ≤ 80% PPO, during which central rather than peripheral fatigue is limiting. Consequently, CHO mouth rinse would be ineffective in improving short-endurance cycling exercise performance with peripheral fatigue as a limiting factor (3).

Studies have reported a growing use of CHO mouth rinse as a strategy to improve performance in different endurance exercise modes such as cycling and running (2, 8, 10, 11). The fact that CHO mouth rinse induces no gastric discomfort, as no ingestion is necessary, may make this strategy a promising ergogenic aid for endurance sports competitions and training sessions (10). In this regard, trainers and cyclists may be interested in knowing if CHO mouth rinse affects outcomes used to prescribe and monitor endurance cycling training sessions, as this may overestimate training zone intensities when compared with non-supplemented conditions. For example, they may want to know if CHO mouth rinse changes the power output and RPE corresponding to the first and second ventilatory thresholds (VT_1_ and VT_2_, respectively) or the PPO associated with the maximal oxygen uptake (i.e., V̇O_2MAX_), as these variables have been traditionally used to determine endurance intensities training zones (12, 13). For example, the ameliorated neuromuscular activation and attenuated RPE with CHO mouth rinse (6) could lead to a higher power output and lowered RPE during exercise at VT_1_ and VT_2_. Unfortunately, studies on CHO mouth rinse effects have focused on cycling time trials (14–17), so we are unaware of studies assessing the effects of CHO mouth rinse on MIT performance and their underlying mechanisms. In contrast to short cycling time trials (8, 9), most of the MIT duration is performed under submaximal intensities-elicited central fatigue (≤80% PPO) (9); thus, one may argue that CHO mouth rinse may potentiate MIT performance due to an ameliorated neuromuscular activation and reduced RPE during most MIT intensities. Consequently, CHO mouth rinse may increase PPO and lower the RPE at VT_1_, VT_2_, and V̇O_2MAX_ intensities during an MIT.

In the present study, we examined if CHO mouth rinse improved MIT performance, changing outcomes such as PPO and RPE at VT_1_, VT_2_, and V̇O_2MAX_ due to attenuated central fatigue, as reported elsewhere (15). Due to submaximal power output values during most stages of the MIT, we hypothesized that CHO mouth rinse would attenuate central fatigue and reduce RPE during exercise, thus improving MIT performance outcomes.

## 2 Materials and methods

### 2.1 Participants

Nine recreational male cyclists ranked as Performance Level 2 (18) (36.9 ± 6.4 years, V̇O_2MAX_ of 54.3 ± 9.3 mL·kg^−1^·min^−1^; body mass of 73.1 ± 13.0 kg; height of 180 ± 5 cm; PPO of 333 ± 29 W), and having >2 years of experience in regional competitions voluntarily participated in this study after signing the consent form. All procedures were approved by the ethics committee of the University of São Paulo (#0023.0.342.000-10) and conducted according to the Declaration of Helsinki. All participants were free from cardiovascular, neural, or musculoskeletal diseases/conditions that could interfere with the experimental protocol. Furthermore, they were asked to refrain from caffeine, alcohol, and other stimulant substances, as well as intense exercises, during the 24 h preceding the experimental tests.

### 2.2 Study design

The cyclists attended the laboratory on four occasions. During the first visit, the cyclists were familiarized with the maximal voluntary contraction (MVC) and twitch interpolation protocol, the 15-point RPE scale (19), and were asked to report their preceding 24-h alimentary intake. On the second visit, they were familiarized with the mouth rinses and performed another familiarization with the MVC before performing a preliminary MIT. During visits 3 and 4, they performed the MIT while rinsing their mouth either with CHO or with a placebo (PLA) solution. Visits 3 and 4 were performed in a randomized, counterbalanced, and double-blind design. Importantly, visits were interspaced by 3–7 days. After performing a 5-min self-paced exercise followed by 1 min controlled-pace exercise (100 W pedaling at 80 rpm), the MIT increased 25 W∙min^−1^ until exhaustion (i.e., the inability to maintain 80 rpm pedal cadence), while strong verbal encouragement was provided by the same researcher. The cyclists used the same speed bicycle (Giant® Thousand Oaks, CA, United States) individually adjusted before each test, connected to a cycle-simulator (Computrainer™ Lab 3D, RacerMate, Seattle, United States) calibrated according to the manufacturer’s recommendation.

### 2.3 Dietary control

We decided to evaluate the cyclists in a fed and normal muscle glycogen state, thus mimicking a condition met in practical situations, as individuals are frequently oriented to consume a balanced diet ~2 h before an MIT in practical situations such as in clinical and sports science environments. Thus, the cyclists followed an individual, one-day alimentary plan (~55% CHO, ~25% protein, and ~20% fat) based on the alimentary record previously provided in the first visit. Then, the cyclists were required to repeat the dietary plan before the experimental sessions, including a breakfast 2 h before the experimental trials. Upon their arrival at the laboratory, we asked them to confirm that they had followed the diet recommendations, mainly the individualized breakfast composed of ~55% CHO, ~25% protein, and ~20% fat.

### 2.4 Mouth rinse

The cyclists rinsed their mouths with 25 mL of CHO or PLA solution for the last 10 s of the controlled-pace warm-up, spitting the substance into a bowl immediately after the mouth rinse (20). The volume spat into the bowl was checked after the test to confirm they had not ingested the solution. Importantly, in addition to the practical approach as above clarified, an MIT is comparable to a high-intensity time trial (9) so that the vigorous body movement and hyperventilation after VT_2_ limit the use of mouth rinse. In contrast, removing the mask of the gas analyzer during the MIT would increase the likelihood of experimental error associated with gaseous exchanges. The CHO solution was composed of 64 g of glucose diluted in 1,000 mL of water (6.4%), while the PLA solution was made of 36.2 g of non-caloric saccharin-based sweetener diluted in 1,000 mL of water (3.6%). These concentrations are in agreement with previous results suggesting that a higher concentration than 6% of CHO produces no increment in exercise performance (21, 22). Importantly, we confirmed that participants were unable to distinguish the placebo from the CHO solution during the familiarization visit.

### 2.5 Twitch interpolation protocol

The pre- and post-knee extension torque measures (twitch torque and interpolated torque) were performed on the dominant limb through a Biodex System 3 (Biomedical Systems ®, Newark, CA, United States) immediately before and after the MIT (<40 s). Participants were seated on the chair, individually adjusted to maintain the hips and knees at 90° and 60°, respectively, from the horizontal plane. Importantly, the chest and hips were carefully fixed to avoid any accessory movements. The estimated center of rotation of the knee joint was visually aligned with the Biodex rotation center. Torque measures were assumed to be the torque produced by the Biodex motor, adjusted by gravity.

To impose supramaximal electrical stimuli, two 7.5 × 13-centimeter self-adhesive electrodes (Mundinter Dantec Clavis ®, Lisbon, Portugal) were positioned on the first- (cathode) and second-thirds (anode) of distance from the anterior superior iliac spine to the proximal border of the patella of the right thigh, respectively. Before the electrode’s placement, regions of the cathode and anode were shaved, abraded, and cleaned using alcohol to reduce skin impedance. Two pulses of 1 ms at 100 Hz were delivered over the muscle belly (Nicolet Viking Quest portable EMG apparatus; CareFusion ®, San Diego, CA, United States) using an individualized intensity determined during the maximum torque at rest (23). To perform the maximal torque test, the participants were instructed to perform a 5-s maximal MVC of the knee extensors. Electric stimuli were imposed at the maximal torque plateau (visually identified) (24), while the second stimulus was imposed 1.5 s after the end of the MVC (potentiated twitch). Torque values were controlled by visual feedback and saved for offline analysis (LabVIEW 2010, National Instruments, São Paulo, Brazil). Voluntary activation (VA) is described by the Equation (1) (25, 26). Delta of peak twitch torque (ΔTw) was measured using the potentiated 100 Hz doublet (after MVC), pre- and post-MIT to determine changes in peripheral fatigue (7, 15).


(1) $$VA\;\left(\%\right)=\left[1-\left(\frac{\mathrm{interpolated}\;\mathrm{twitch}}{\mathrm{potentiated}\;\mathrm{twitch}}\right)\right]*100$$

### 2.6 Measures and data analysis

#### 2.6.1 Maximal incremental test performance

Time-to-exhaustion, PPO, and power output at VT_1_ and VT_2_ expressed as absolute and relative values (% PPO) were used as performance outcomes.

#### 2.6.2 Cardiopulmonary responses

Cardiopulmonary responses such as oxygen uptake (V̇O_2_) and minute ventilation (V̇E) were assessed breath-by-breath through an open-system gas analyzer (Cosmed, Quark PT, Albano Laziale, Rome, Italy) calibrated before each test. Expired air volume was assessed through a bi-directional flow sensor calibrated before each test using a 3-L air syringe. The cyclists used a mask (Hans Rudolph R, Lenexa, KS, United States) connected to the gas analyzer to measure breath-by-breath gaseous exchange, which was filtered to 10 s epochs thereafter. V̇O_2MAX_ was calculated as the average of the last 30 s of the test (27). Moreover, two evaluators visually identified VT_1_ and VT_2_ at the first and second breakpoints of the V̇E–time relationship, respectively. These breakpoints were confirmed by the breakpoint of the expired fractions of O_2_ (FeO_2_) and CO_2_ (FeCO_2_) responses (27). The PPO was calculated as suggested by De Pauw et al. (18) (Equation 2), where PO1 is the power output during the last fully completed stage, PO2 is the power output during the highest stage (not completed), T1 is the time (in min) of the previous stage duration, and T2 is the time spent at the last stage.


(2) $$PPO=PO1+\frac{\left[\left(PO2-PO1\right)T2\right]}{T1}$$

#### 2.6.3 Ratings of perceived exertion

The RPE was assessed at the end of each MIT stage so that the RPE corresponding to VT_1_ and VT_2_ were reported. In addition, the slope of the RPE–time relationship (RPE_SLOPE_) was used to verify how cyclists’ RPE increased throughout the MIT after CHO or PLA mouth rinse.

### 2.7 Statistical analysis

Data normality and homoscedasticity were initially checked through the Shapiro–Wilk and Levene tests, respectively. Outcomes such as V̇O_2MAX_, PPO, time-to-exhaustion, and RPE_SLOPE_ were compared using a paired Student’s t-test (CHO vs. PLA). Absolute and relative values of PPO and V̇O_2_ (% PPO or % V̇O_2MAX_) and RPE at VT_1_ and VT_2_ were compared through a 2 × 2 mixed model having time (VT_1_ vs. VT_2_) and treatment (CHO vs. PLA) as fixed factor, while subjects were the random factor. Accordingly, VA and Tw values obtained pre- and post-MIT were compared through a 2 × 2 mixed model having time (pre- vs. post-MIT) and treatment (CHO vs. PLA) as fixed factors, while the subjects were the random factor. Multiple comparisons were corrected through Bonferroni’s test when *F* values were significant. Effect size (ES) was calculated according to the family test used, expressed as Cohen’s *d*, to make possible comparisons with previous studies. Moreover, ES values were interpreted as proposed by Hopkins et al. (28) as Cohen’s *d* ≤ 0.1 is considered small, 0.1> and <0.3 moderate, 0.3> and <0.5 large, 0.5> and <0.9 very large, and >0.9 extremely large. Statistical significance was set at 5% (*p* < 0.05).

## 3 Results

### 3.1 MIT performance and outcomes

There was no significant difference in V̇O_2MAX_ (t = 0.552, *p* = 0.60, d = 0.10, ES small), PPO (t = −0.785, *p* = 0.45, d = 0.08, ES small), and time-to-exhaustion (t = −1.259, *p* = 0.24, d = 0.12, ES small) between CHO and PLA mouth rinse. RPE increased throughout the MIT similarly in both CHO and PLA mouth rinse conditions, given the comparable RPE_SLOPE_ between conditions (t = −0.610, *p* = 0.56, d = 0.19, ES = moderate).

As expected, responses at VT_1_ were different from VT_2_; thus, we omitted time main effects from the manuscript to make the reading easier. There was no treatment main effect, so absolute V̇O_2_ (*F* = 0.08, *p* = 0.78, d = 0.15, ES small) and power output values (*F* = 1.33, *p* = 0.28, d = 0.58, ES large), as well as % V̇O_2MAX_ (*F* = 0.003, *p* = 0.96, d = 0.03, ES small) and % PPO (*F* = 0.64, *p* = 0.45, d = 0.40, ES large), were comparable between CHO and PLA conditions. Additionally, no treatment-by-time interaction effect was found either in absolute V̇O_2_ (*F* = 0.10, *p* = 0.76, d = 0.17, ES small) and power output values (*F* = 0.26, *p* = 0.62, d = 0.26, ES moderate) or in % V̇O_2MAX_ (F = 0.003, p = 0.96, d = 0.03, ES small) and % PPO values (*F* = 0.31, *p* = 0.58, d = 0.28, ES moderate). Accordingly, neither treatment main effect (*F* = 1.13, *p* = 0.31, d = 0.53, ES = large) nor time-by-treatment interaction effect (*F* = 0.79, *p* = 0.40, d = 0.44, ES = large) was observed in RPE. All these MIT outcomes were reported in Table 1.

**Table 1:** Performance and outcomes assessed in the maximal incremental test.

	Carbohydrate	Placebo
V̇O_2MAX_ (ml∙kg^−1^∙min^−1^)	51.9 ± 7.0	51.2 ± 5.3
V̇O_2_ (ml∙kg^−1^∙min^−1^) at VT_1_	33.5 ± 3.1	33.4 ± 3.6
V̇O_2_ (ml∙kg^−1^∙min^−1^) at VT_2_	48.2 ± 7.0	47.6 ± 4.8
V̇O_2_ (%V̇O_2MAX_) at VT_1_	60.4 ± 8.0	60.4 ± 5.0
V̇O_2_ (%V̇O_2MAX_) at VT_2_	85.8 ± 3.8	85.9 ± 3.1
PPO (W)	328.8 ± 42.0	332.3 ± 42.2
PO (W) at VT_1_	170.8 ± 21.6	183.3 ± 30.0
PO (W) at VT_2_	283.3 ± 35.9	289.0 ± 36.1
PO (% PPO) at VT_1_	52.2 ± 5.5	55.2 ± 6.8
PO (PPO) at VT_2_	86.3 ± 6.0	87.2 ± 6.4
Time to exhaustion (s)	610.4 ± 99.1	622.4 ± 94.8

Results were expressed as mean ± SD.

### 3.2 Voluntary activation and twitch interpolation responses

**Figure 1:**
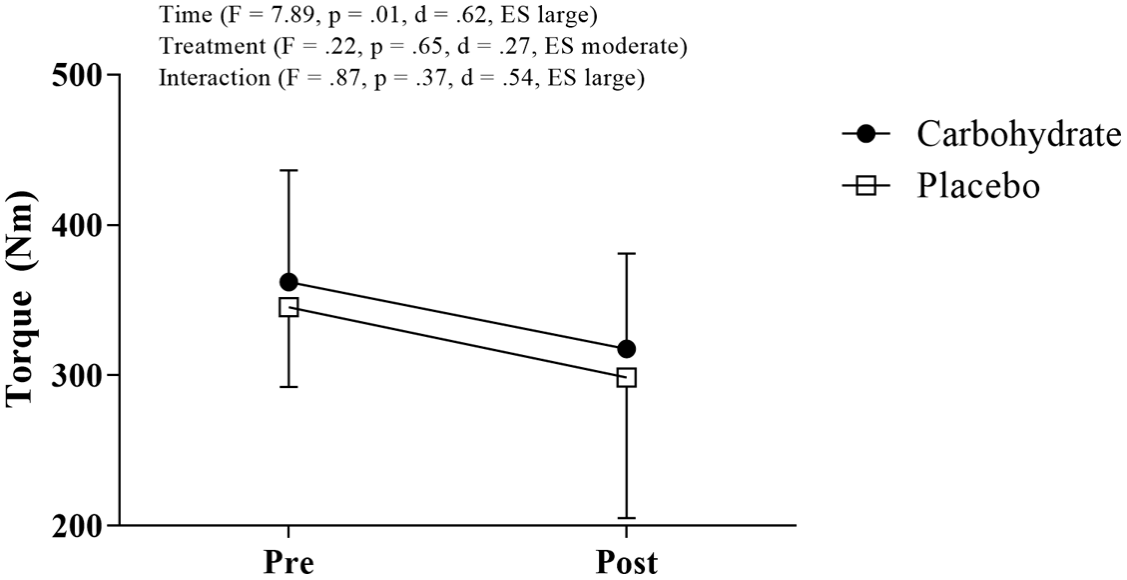
Torque measured before and after MIT in carbohydrate (black circle) and placebo (white square) mouth rinse trials.

There was neither the time-by-treatment interaction effect (*F* = 0.87, *p* = 0.37, d = 0.54, ES large) nor the treatment main effect (*F* = 0.22, *p* = 0.65, d = 0.27, ES moderate); however, a time main effect (*F* = 7.89, *p* = 0.01, d = 0.62, ES large) was detected on torque responses, indicating an exercise-induced reduction (Figure 1).

**Figure 2:**
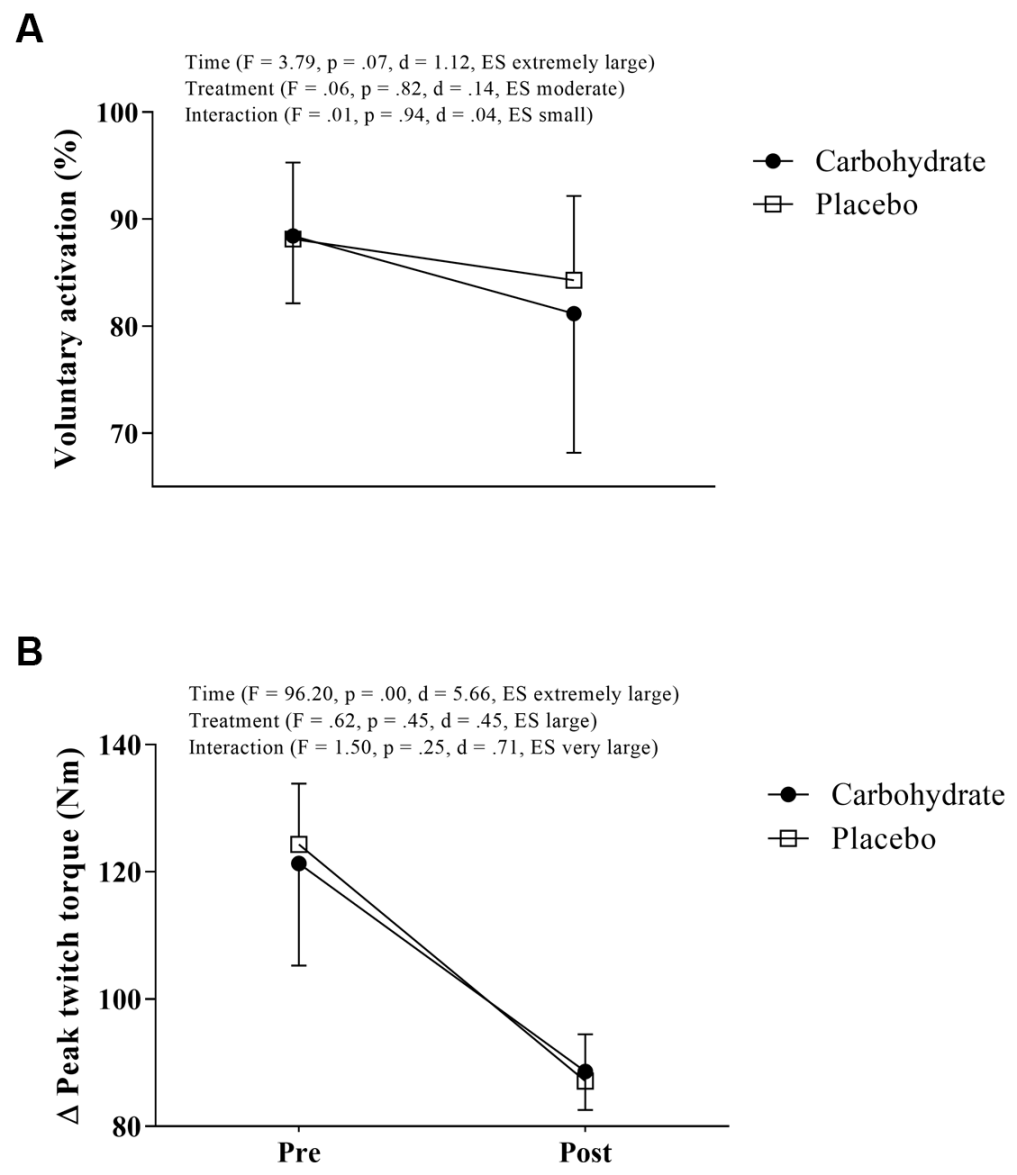
Voluntary activation **(A)** and peak twitch torque **(B)** measured before and after MIT in carbohydrate (black circle) and placebo (white square) mouth rinse trials.

Accordingly, neither the time-by-treatment interaction effect (*F* = 0.01, *p* = 0.94, d = 0.04, ES small) nor the treatment main effect (*F* = 0.06, *p* = 0.82, d = 0.14, ES moderate) was observed in VA, despite the borderline value of p and extremely large ES (*F* = 3.79, *p* = 0.07, d = 1.12, ES extremely large) suggesting an exercise-reduced VA (i.e., time main effect). Similarly, neither the time-by-treatment interaction effect (*F* = 1.50, *p* = 0.25, d = 0.71, ES very large) nor the treatment main effect (*F* = 0.62, *p* = 0.45, d = 0.45, ES large) were observed in ΔTw; however, the time main effect in ΔTw (*F* = 96.20, *p* < 0.01, d = 5.66, ES extremely large) evidenced exercise-increased peripheral fatigue. Figures 2A,B show central and peripheral fatigue responses.

## 4 Discussion

We investigated if MIT outcomes may be improved with CHO mouth rinse due to reduced central fatigue and RPE. In agreement with previous results in short-cycling exercises (8, 29), we found that CHO mouth rinse did not improve MIT outcomes such as VT_1_, VT_2_, PPO, and V̇O_2MAX_, although moderate-to-large effect sizes may suggest some beneficial effects in VT_1_ and VT_2_ outcomes. Despite the moderate-to-large effect size, we also found null effects of CHO mouth rinse in the central fatigue index, as reported elsewhere (8, 15). These results provide support to suggest that CHO mouth rinse has neglectable beneficial effects to reduce central fatigue and improve performance outcomes in MIT (8).

Several studies have suggested that CHO mouth rinse may improve different performance outcomes (3, 30), despite some null effects reported elsewhere (8, 14, 31). An earlier study showed improvements in ~1 h cycling time trial performance with CHO mouth rinse when participants were evaluated in fasted (↑ 3.4%) as well as fed state (↑ 1.8%), although the lower effect was in the fasted state (32). Thus, we used a logic-driven hypothesis to expect some improvement in MIT performance even though participants were in a fed state. In brief, we evaluated participants in a fed state to mimic practical situations from sports scenarios, as individuals are usually recommended to consume a balanced diet ~2 h before an MIT. Our results showed that CHO mouth rinse was ineffective at improving MIT performance outcomes such as time-to-exhaustion, PPO, and power output at VT_1_ and VT_2_. Consequently, no changes were observed in V̇O_2_ responses such as V̇O_2MAX_ and V̇O_2_ at VT_1_ and VT_2_. Furthermore, PLA and CHO also showed comparable RPE responses throughout the MIT. These null results relate to the mechanisms suggested for the CHO mouth rinse ergogenic aid and to the exercise mode used in the present study.

Earlier studies by Chambers et al. (3) and Turner et al. (4) suggested that the presence of CHO in the oral cavity may increase the activation in motor planning cerebral areas associated with the reward circuit system and exercise regulation (33). Chambers et al. (3) showed that those cyclists who improved their time-trial performance at the same RPE in study 1 (greater average power output and reduced elapsed time in the last 75% of the cycling trial) elicited a further enhanced cerebral activation in frontal and premotor areas in study 2. Thus, they proposed that the enhanced activity in areas related to reward and exercise regulation in study 2 explained the improved performance and reduced RPE during exercise in study 1 (3, 4, 33). This mechanism may be potentiated in the participants in a fasted rather than fed state. Studies have observed that variations in the food-derived metabolic signals such as plasma ghrelin and leptin may play a role in the CHO mouth rinse-induced modulation in cerebral activity, as this signalization may induce greater cerebral responses mainly in periods of food abstinence longer than 4 h (10). Consequently, CHO mouth rinse-increased cerebral activation may not have been optimal in the present study, as participants were assessed in a fed state. Furthermore, the fact that the participants were allowed to have CHO meals while avoiding intense exercises during the 24 h before the experimental sessions likely preserved their muscle glycogen stores upon their arrival at the laboratory. This fact may have minimized the impact of an eventual improved cerebral activation due to the CHO mouth rinse, thus contributing to the null effects observed in MIT performance.

The exercise mode used in the present study may also have minimized the ergogenic aid effect of the CHO mouth rinse. We found no CHO mouth rinse effect on exercise-induced central fatigue, as reductions in VA after MIT were comparable between CHO (↓ ~3.01%) and PLA (↓ ~4.14%) mouth rinses. Similarly, RPE responses were comparable between both mouth rinses. CHO mouth rinse may reduce RPE and potentiate performance in long rather than short-term exercises, as the former is known to induce considerable levels of central fatigue (7). Thomas et al. (7) observed that short-lasting time trials such as 4 km induced greater peripheral fatigue as measured by potentiated quadriceps twitch force (i.e., ∆Tw) while longer-lasting ones (20 and 40 km) induced greater central fatigue. Hence, considering the central action of CHO mouth rinse, this strategy may be effective in attenuating central fatigue (34) and potentiating performance in cycling exercises longer than MIT exercises. Interestingly, we found that CHO mouth rinse failed to potentiate a 4-km cycling time trial despite the reduced central fatigue (8). Considering these exercise modes induce comparable physiological responses mainly from VT_2_ intensities (9), the ineffectiveness of the CHO mouth rinse to improve MIT outcomes may be possibly related to the fact that peripheral rather than central mechanisms limit performance in this exercise mode.

### 4.1 Methodological aspects and practical implications

Some methodological aspects should be highlighted. First, we are aware that neuromuscular fatigue recovers as soon as the exercise has been terminated so the time taken to obtain neuromuscular measures after the MIT may be a concern. However, we rehearsed the transition from the bike to the isokinetic dynamometer several times, which greatly decreased the transition time (~40 s). Although we may have missed some of the immediate changes post-MIT, in our experience, this time window is sufficient to detect neuromuscular changes as those reported in the literature (7, 8).

Most CHO mouth rinse studies used rinses at regular intervals, frequently at every 12.5% of the test duration, when investigating its effects on exercise performance (3, 11, 35). In contrast, in the present study, the participants rinsed their mouths once during the last 1 min of the cycling warm-up. This strategy was used for different reasons. First, the use of mouth rinse during an MIT, mainly from VT_2_ intensity, could preclude participants from the maximal effort, as they would have to rinse their mouth and spit the solution out while hyperventilating. Furthermore, as the gaseous exchange was assessed throughout the MIT, the mouth rinse would require removing the gas analyzer mask during exercise, thus increasing the likelihood of error on the gaseous exchange measure. Hence, considering the fact that CHO mouth rinse effects seem to be timely (6), the use of a single 10-s mouth rinse before the MIT commencement is apparently ineffective in improving MIT outcomes.

Moreover, despite controversial results indicating positive (32, 36) and null effects of CHO mouth rinse in both fed and fasted states (29, 37), Lane et al. (32) suggested that the magnitude of the CHO mouth rinse effect may be reduced when the subjects are fed. This suggestion challenges the utility of this strategy to potentiate exercise performance in clinical and sports sciences scenarios, as athletes and exercise practitioners are frequently recommended to avoid a fasted state when exercising (38). A systematic review found that the post-prandial state did not moderate the CHO mouth rinse effects on exercise performance (2), thus indicating that muscle glycogen availability may be the primary factor influencing the ergogenic benefit of CHO mouth rinse. Therefore, future studies are necessary to reveal if rinsing the mouth with CHO solution effectively reverses a depleted muscle glycogen-reduced MIT performance.

Anecdotal data suggest a growing use of this strategy to improve performance in different endurance sports, such as cycling (8). The fact that CHO mouth rinse neither induces gastric discomfort during exercise (as no ingestion is necessary) nor belongs to the banned substances list, according to the World Anti-Doping Agency (WADA), may put this strategy as a promising ergogenic aid in endurance sports scenarios. Consequently, trainers, coaches, and cyclists may be interested in knowing if CHO mouth rinse really influences MIT outcomes used to prescribe and monitor endurance cycling training sessions such as power output and RPE at VT_1_ and VT_2_ intensities. The results of the present study challenged the use of MIT with this purpose, although some clarification should be addressed at this moment. In brief, we did not calculate the sample size required to detect likely CHO mouth rinse effects according to the most probable effect size. In an earlier study (2), we calculated that CHO mouth rinses had only a small, despite significant effect size on cycling performance when expressed as Hedge’s coefficient (g = 0.25; *p* = 0.02), so that a very large and unrealistic sample size of 128 participants would be necessary to detect an eventual beneficial effect on cycling exercise performance in a paired T-test design as used in the present study. The *post-hoc* calculations revealed only a small CHO mouth rinse effect size on maximal parameters such as PPO and time-to-exhaustion. However, the moderate-to-large effect sizes found in power output values at VT_1_ and VT_2_ suggest that a sample size with nine participants was probably underpowered to detect beneficial CHO mouth rinse effects in these submaximal performance parameters. Therefore, despite the methodologically sound challenge of the use of CHO mouth rinse in MIT outcomes such as PPO and time-to-exhaustion, future studies with greater sample sizes are required to confirm the effectiveness of this nutritional strategy in VT_1_ and VT_2_ in participants in a fed state.

## 5 Conclusion

Our results showed that rinsing the mouth with CHO before commencing an MIT was ineffective in reducing central fatigue, lowering the RPE, and improving MIT performance expressed as PPO and time-to-exhaustion. However, moderate-to-large effect sizes in power output values at VT_1_ and VT_2_ may suggest some beneficial CHO mouth rinse effects on these MIT outcomes.

## Data availability statement

All relevant data are within the paper. The raw data of this article may be made available by request.

## Ethics statement

The studies involving humans were approved by Ethical Committee of the University of São Paulo (#0023.0.342.000-10). The studies were conducted in accordance with the local legislation and institutional requirements. The participants provided their written informed consent to participate in this study.

## Author contributions

FOP: Conceptualization, Data curation, Formal Analysis, Funding acquisition, Methodology, Writing – original draft, Writing – review & editing. FAP: Formal Analysis, Investigation, Writing – review & editing. CB: Data curation, Formal Analysis, Investigation, Writing – original draft. PF-A: Data curation, Formal Analysis, Investigation, Writing – original draft. KV: Formal Analysis, Investigation, Writing – original draft. EM: Conceptualization, Investigation, Writing – original draft, Writing – review & editing. AR: Conceptualization, Project administration, Supervision, Writing – review & editing. CU: Conceptualization, Resources, Supervision, Writing – review & editing.
